# Geschichte der klinischen Elektrophysiologie in Diagnostik und Therapiekontrolle ventrikulärer Vulnerabilität

**DOI:** 10.1007/s00399-024-01005-1

**Published:** 2024-02-27

**Authors:** Dietrich Pfeiffer

**Affiliations:** Berlin, Deutschland

**Keywords:** Geschichte der Kammervulnerabilität, Plötzlicher Herztod, Elektrophysiologie zur Therapiekontrolle, History of ventricular vulnerability, Prediction of sudden cardiac death, Electrophysiology for treatment control

## Abstract

Der Beitrag beschreibt die Geschichte einer Hoffnung in den Jahren 1980 bis 1995, das Risiko eines plötzlichen arrhythmischen Herztodes mit den Methoden der klinischen Elektrophysiologie am einzelnen Patienten ausreichend sicher vorherzusagen. Auch wenn eine solche Wahrscheinlichkeit in ausgewählten Gruppen mit hoher Zuverlässigkeit bestimmt werden kann, so entziehen sich viele klinische Szenarien einer solchen Vorhersage. Letztlich haben umfangreiche Bemühungen nur eine statistische Gruppenwahrscheinlichkeit, nicht eine therapierelevante Wahrscheinlichkeit im Einzelfall ergeben. Es ist die Geschichte einer enttäuschten Hoffnung.

## Abstract (en)

The paper presents the history of hope from 1980–1995 to predict the risk of sudden arrhythmic death using electrophysiologic techniques in individual patients. Even if this prediction seems possible in selected highly risk cohorts, many more patients will die in ventricular arrhythmia without fulfilling the criteria. Ultimately, high risk of sudden cardiac death can be predicted in selected patient groups, but not in the majority of patients at risk. It is a history of dashed hope.

Der plötzliche Herztod wurde seit Mitte des 20. Jahrhunderts als häufiges, zu Reanimation oder gar zum Tode führendes Ereignis beschrieben. Oftmals sind ältere und bekanntermaßen herzkranke Patienten betroffen, nicht selten tritt ein solches Ereignis aber auch bei jüngeren, bislang nicht identifizierten Herzkranken auf. Erfolgt nach einem Notfall eine kardiologische Diagnostik oder gar eine Obduktion, so finden sich manchmal bislang asymptomatische koronare, valvuläre, entzündliche oder myokardiale Erkrankungen. Nicht selten findet sich aber keinerlei Hinweis auf eine Herzerkrankung. Die sporadische Erfassung eines plötzlichen Todesfalls durch zufällige EKG-Registrierung, durch Langzeit-Elektrokardiographie [1], während Belastungsuntersuchungen oder unter Monitorüberwachung belegte, dass in der überwiegenden Zahl (84 %) der Betroffenen eine ventrikuläre Tachyarrhythmie vorliegt [2] (Abb. 1). Diese klinische Ausgangssituation war seit Jahrzehnten gesichert.

**Figure 1:**
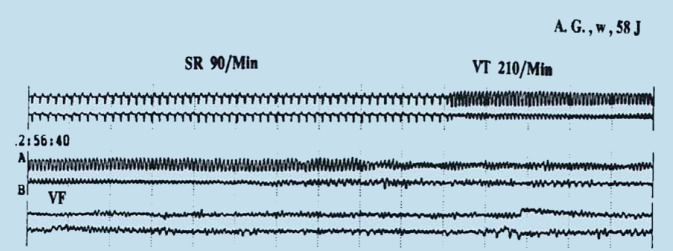

Daraus ergab sich das Problem, gefährdete, jedoch bislang asymptomatische, mithin nicht identifizierte Risikoträger zu erkennen, weil bereits ihr erstes klinisches Symptom eine lebensbedrohende Arrhythmie sein kann. War das Risiko bei Überlebenden eines plötzlichen Herztodes durch das Ereignis gesichert, wurden die Grunderkrankung behandelt, und es wurde versucht, die offensichtlich erhöhte ventrikuläre Vulnerabilität mit Antiarrhythmika zu unterdrücken. Bei potenziell proarrhythmischen Medikamenten war anschließend die erfolgreiche Suppression der ventrikulären Vulnerabilität zu belegen und die Gefahr einer Proarrhythmie auszuschließen. Mit diesen Zielen wurden zahllose Elektrokardiogramme geschrieben, die zumeist keine spontanen Kammertachykardien enthielten. Am häufigsten, aber auch nicht regelhaft, ließen sich ventrikuläre Extrasystolen nachweisen, mit deren Unterdrückung unter Antiarrhythmika parallel eine Suppression von Tachyarrhythmien unterstellt wurde. Es war Bernard Lown, der eine klinische Graduierung von Extrasystolen mit geringem oder höherem Risiko eines plötzlichen Herztodes vorschlug. Extrasystolen wurden als prämonitorische Warnarrhythmien für ein Kammerflimmern verstanden [3]. Wenn unter antiarrhythmischer Medikation eine Abnahme der Anzahl von Extrasystolen oder in der Graduierung nach Lown zu verzeichnen war, dann fühlten sich Arzt und Patient ausreichend sicher. Es gab jedoch zu allen Zeiten Zweifel, ob diese Logik eine klinische Bestätigung findet. Nur gab es keine andere Möglichkeit der Therapiekontrolle. Bessere Optionen der Vulnerabilitäts- und Wirksamkeitsprüfung von Antiarrhythmika gab es vor Entwicklung der klinischen Elektrophysiologie nicht.

Diese Beobachtungsstrategie spontaner Rhythmusstörungen hatte von Anbeginn zwei wesentliche Einschränkungen: Die stark wechselnde Häufigkeit der Arrhythmien führte dazu, dass oftmals unauffällige Aufzeichnungen über lange Zeiträume nachgewiesen wurden, jedoch der Patient dennoch unerwartet unter einer ventrikulären Tachyarrhythmie verstarb. Hatte man sich zur antiarrhythmischen Behandlung entschlossen, so war manchmal weder eine Proarrhythmie noch ein Behandlungserfolg mit ausreichender Sicherheit zu belegen. Wurden Extrasystolen registriert, so wurde von prämonitorischen Arrhythmien ausgegangen, die es zu unterdrücken galt, und es wurde im Fall einer Reduktion der Extrasystolen eine ausreichende Behandlung der Kammertachykardie unterstellt. Manchmal trat die Rhythmusstörung dennoch bereits kurze Zeit später wieder auf.

Die Situation änderte sich, als Hein JJ Wellens beschrieb, dass viele ventrikuläre Tachyarrhythmien mit programmierter Elektrostimulation initiierbar waren [4]. Es lag nahe, mittels programmierter Stimulation zumindest die initial reproduzierbar auslösbaren ventrikulären Tachykardien zu induzieren und diesen Befund unter Antiarrhythmika mit denselben Stimulationsalgorithmen zu kontrollieren. Dieses Konzept sah in der Langzeitregistrierung nur noch die Kontrolle der Initiierungsmechanismen (Extrasystolen, Salven, nichtanhaltende ventrikuläre Tachykardien, intermittierende Bradykardien) und in der programmierten Stimulation die Kontrolle der Perpetuierung der Arrhythmie. Im Idealfall einer ursprünglich auslösbaren monomorphen Tachykardie und reichlich komplexen Extrasystolen, jedoch nach Behandlung mit fehlender Auslösbarkeit und fehlenden Extrasystolen im Langzeit-EKG unter Medikamentenwirkung hielten wir den Patienten für ausreichend sicher behandelt [3].

Das Konzept der Initiierung ventrikulärer Tachykardien mit programmierter Stimulation begann mit ethischen Diskussionen: Konservative alte Internisten lehnten das Konzept gelegentlich als unethische Gefährdung der Patienten ab, die man doch nicht in eine lebensbedrohende Situation bringen dürfe. Unsere Arbeitsgruppe in Berlin wandte sich an einen älteren erfahrenen allgemeininternistischen Chefarzt, der nach eingehender Diskussion potenzieller Gefahren für den Patienten das Konzept als schlüssig und vertretbar betrachtete und uns zuriet, mit der gebotenen Vorsicht weiterzumachen.

Es folgte eine jahrelange Diskussion um Stimulationsalgorithmen: Mit welchen Basisfrequenzen (mindestens 2–3), wieviel Extrastimuli (maximal 3), Bursts (bis zu einer Zyklusdauer von 200 ms), „incremental pacing“, Short-long-short-Sequenzen, bis zu welchem minimalen Kopplungsintervall (≥ 200 ms), mit linksventrikulärer Stimulation, unter Medikation (z. B. Isoprenalin), zu welcher Tageszeit, mit welchem Abstand nach gesichertem Myokardinfarkt (≥ 4 Wochen) durfte die elektrophysiologische Untersuchung erfolgen? Natürlich nahm mit zunehmender Aggressivität einer Stimulation die Wahrscheinlichkeit nichtklinischer, mithin falsch-positiver Tachykardie-Initiierung zu.

Rasch erwiesen sich aber bei der Prüfung der elektrophysiologischen Stimulation manche spontan aufgetretenen Kammerarrhythmien als nicht auslösbar. Dies betraf nicht nur fokal-automatische oder getriggerte Tachykardiemechanismen und Kammertachykardien bei Repolarisationssyndromen, bei denen Nichtinduzierbarkeit geradezu zu erwarten war, sondern auch eindeutige Reentry-Tachykardien bei Kardiomyopathien, chronischer Myokarditis, erheblichen Hypertrophien, dysplastischen Erkrankungen, ischämiegetriggerten Kammertachykardien oder Kanalopathien, manchmal selbst bei Postinfarktpatienten. Auch bei Patienten mit scheinbar gesundem Herzen, die eigentlich wegen atrialer oder atrioventrikulärer Rhythmusstörungen oder gar wegen Bradykardien untersucht wurden, wurden sporadisch Kammertachykardien ausgelöst. Schließlich ließ sich nachweisen, dass das aktuelle Ausmaß der Myokardischämie, der Volumenstatus des Kreislaufs, eine Elektrolytimbalance und vor allem die Aktivität des autonomen Nervensystems Einfluss auf die Initiierbarkeit von Arrhythmien haben. Die Interpretation mancher Befunde der Elektrophysiologie blieb schwierig: Konnte man wirklich die Überführung einer zuvor anhaltenden Tachykardie in eine nichtanhaltende Tachykardie < 30 s Dauer als ausreichenden Therapieerfolg werten? Würde sich eine Verlängerung der Tachykardiezyklusdauer unter Antiarrhythmika auch klinisch als ausreichend erweisen? War die Verkürzung einer ausgelösten nichtanhaltenden Tachykardie von 10 s Dauer auf nur noch 5 s Dauer ein ausreichend gutes Ergebnis? Solche Fragen wurden auf zahlreichen Tagungen diskutiert, und es kam zu einer Vielzahl von Studien zu diesen Fragen. Klinische Zweifel in der Interpretation der Befunde blieben.

Georg Heinrich v. Knorre, der als einer der Gründerväter der klinischen Elektrophysiologie auf der östlichen Elbseite gilt, sagte uns auf einer Tagung in den 70er Jahren: „Ich bekomme jeden Patienten ins Kammerflimmern. Notfalls stecke ich den Katheter in die Steckdose.“ Das hat er wohl nie gemacht, aber dieses Wort zeigte schon damals Grenzen des Konzepts.

Die Ergebnisse mehrerer vergleichender Studien zwischen Holter-Monitoring und programmierter Stimulation waren nicht identisch. Brugada berichtete über eine Kontrolle der Antiarrhythmika-Wirkung mit elektrophysiologischen Techniken und Holter-Monitoring in der „Parallel study“ an 84 Postinfarktpatienten mit ventrikulären Tachykardien (*n* = 64) oder Kammerflimmern (*n* = 20) [5]. Alle Arrhythmien waren initiierbar, jedoch nur bei 29 Patienten (34,5 %) fanden sich im Langzeit-EKG ventrikuläre Arrhythmien im Schweregrad Lown IVb. Damit zeigte die Registrierung von spontanen Arrhythmien ein falsch-negatives Ergebnis der Arrhythmieneigung von 66 %. Unter einer Antiarrhythmika-Therapie waren 68 Tachyarrhythmien (81 %) unverändert induzierbar, und bei 8 der 29 Patienten (27,6 %) mit ausreichend Arrhythmien im Langzeit-EKG blieb der Arrhythmieschweregrad nach Lown identisch. Über 16 Monate Follow-up kam es bei 25 von 68 der Patienten mit induzierbaren Tachykardien auch spontan zu erneuten Tachykardien im Vergleich zu 8 Patienten (12,5 %), die trotz fehlender Induzierbarkeit ein Rezidiv entwickelten. Dennoch kam es bei 63 % der Patienten trotz unveränderter Induzierbarkeit nicht zu einem spontanen Rezidiv. Umgekehrt wurden jedoch bei 13 % trotz fehlender Induzierbarkeit spontane Rezidive beobachtet. Offenbar waren beide Techniken nicht sicher in der prognostischen Bewertung.

Eine weitere vergleichende Untersuchung von programmierter Stimulation versus Holter-EKG in der Kontrolle mit Antiarrhythmika an 57 Patienten wurde 1987 mitgeteilt [6]. Als Kriterien einer erfolgreichen Suppression wurden für beide Methoden strengere Grenzen festgelegt: Im Holter-EKG mussten als Therapieerfolg spontane Extrasystolen um > 90 % reduziert und Salven von 3 und mehr konsekutiven Aktionen vollständig unterdrückt sein. Unter Stimulation galt die fehlende Auslösbarkeit anhaltender Tachykardien bis zu 5 konsekutiven Extrasystolen als Erfolg. Unter diesen Kriterien galten alle Patienten in der Spontanregistrierung von Arrhythmien als erfolgreich behandelt, während es mittels programmierter Stimulation nur 53 % waren. Allerdings entwickelten im Verlauf von 2 Jahren 20 % der mittels programmierter Stimulation kontrollierten Arrhythmien und 50 % der mit Holter-Monitoring nachverfolgten Patienten Rezidive. In der Diskussion wurde angemerkt, dass die programmierte Stimulation einen höheren prädiktiven Wert im Vergleich zum Langzeit-EKG besitzt. In der Konsequenz wurden beide Methoden gemeinsam für die Therapiekontrolle empfohlen.

Schließlich konnten die Stimulationskonzepte beim Postinfarktpatienten bei anderen Grunderkrankungen, wie z. B. bei hypertropher Kardiomyopathie (HCM), nicht einfach übernommen werden. Während sich bei Patienten mit HCM nach Synkopen oder Reanimationen mit 66 bis 100 % häufig ventrikuläre Tachykardien induzieren ließen im Vergleich zu asymptomatischen Patienten mit HCM, kam es bei nichtinduzierbaren Tachyarrhythmien im Verlauf doch zu plötzlichen Todesfällen. Auch ließ sich keine Beziehung zu morphologischen oder hämodynamischen Parametern nachweisen [7]. Bei Patienten mit dilatativer Kardiomyopathie (DCM) ohne Synkope oder Reanimation konnten bis 13 % anhaltende ventrikuläre Tachyarrhythmien ausgelöst werden, deren klinische Relevanz unsicher blieb. Die Angaben zur Sensitivität der programmierten Stimulation in der Vorhersage des plötzlichen Herztodes bei DCM schwankten zwischen 0 und 45 % [8].

Damit waren prospektive und randomisierte Studien nötig. In der Therapiekontrolle einer medikamentösen Behandlung von ventrikulären Tachykardien belegte die ESVEM-Studie eine vergleichbare prognostische Sicherheit beider Methoden hinsichtlich Arrhythmierezidiv oder plötzlichem Tod [9]. In der Diskussion der Veröffentlichung wurde elektrokardiographisches Monitoring der elektrophysiologischen Testung vorgezogen, weil eine erfolgreiche antiarrhythmische Suppression häufiger nachzuweisen war und weil die Methode weniger zeitintensiv, nichtinvasiv, den Patienten weniger belastend und kostenintensiv sei. Methodenkritisch angemerkt wurde, dass in ESVEM ausschließlich Patienten mit induzierbaren Kammertachykardien eingeschlossen wurden, so dass ein Vergleich mit einer unselektionierten Patientengruppe nicht möglich ist. Hinzu kommt, dass nicht bei allen Patienten im Langzeit-EKG genügend Extrasystolen registriert wurden, um einen suppressiven Effekt zu belegen. Außerdem gab es in beiden Gruppen Rezidive und Todesfälle in der optimal behandelten Patientengruppe von 20 % (elektrophysiologisch untersuchte Patienten) versus 50 % (Patienten mit Holter-Monitoring).

Solange es keine definitive Behandlung von Kammertachykardien mittels antiarrhythmischer Chirurgie oder Katheterablation oder zumindest die rasche und sichere Terminierung mit implantierbarem Kardioverter-Defibrillator (ICD) gab, wurde das Konzept einer Kombination von Spontanregistrierung von Arrhythmien und programmierter Stimulation nativ und unter antiarrhythmischer Medikation fortgesetzt. Die Situation änderte sich mit zunehmender Verfügbarkeit von ICDs zu Beginn der 80er Jahre erneut: Nun stand die Frage, wer denn den ICD bekommen sollte? Da die Geräte anfangs teuer, die Implantation eine mediane Sternotomie und epikardiale Platzierung von Patch-Elektroden erforderte und nachfolgende Probleme (Heilungsstörungen, Infektionen, Patch-Migration, inadäquate Schocks) nicht ganz selten auftraten, wurde die Indikation zunächst begrenzt. Diese Einschränkungen führten dazu, dass Hochrisikogruppen von Patienten zu identifizieren waren, die nach Implantation eine besonders hohe Wahrscheinlichkeit hatten, dass das Gerät absehbar auch adäquat intervenieren würde. Der Patient nach Myokardinfarkt mit hochgradig eingeschränkter Pumpfunktion, klinischer Herzinsuffizienz, nach überlebtem plötzlichem Herztod und initiierbarer Kammertachykardie war der typische ICD-Patient der ersten Jahre. Es wurden Defibrillationsschwellen und später auch tachykardiepräventive und -terminierende Stimulationsalgorithmen programmiert und untersucht. Es zeigte sich rasch, dass die identifizierten Hochrisikogruppen in der Tat eine hohe Wahrscheinlichkeit aufwiesen, eine adäquate Schockabgabe zu benötigen. Aber die Hochrisikogruppe schloss nur eine kleine Subgruppe aller der Patienten ein, die im Verlauf wirklich einen plötzlichen Herztod erlitten: Viel mehr Patienten gehörten jedoch nicht zur Hochrisikogruppe, verstarben aber trotzdem am plötzlichen Herztod (Abb. 2). Wollte man die Mehrzahl aller gefährdeten Patienten erfassen, so half die klinische Elektrophysiologie mit der programmierten Stimulation wenig und wurde deshalb zunächst bei folgenden klinischen Studien [10], dann aus Editorials und Übersichtsarbeiten [11] und schließlich aus den empfohlenen Behandlungsalgorithmen [12] eliminiert.

**Figure 2:**
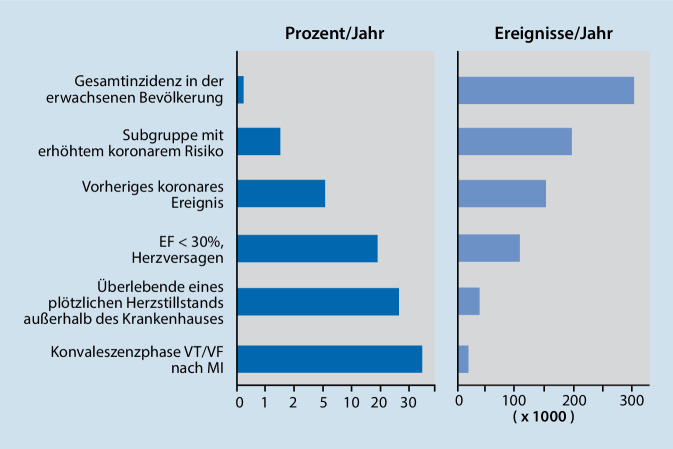

Die Vorhersage des plötzlichen Herztodes stand einige Jahre im Mittelpunkt des wissenschaftlichen Interesses der Kardiologen. Das führte zu teilweise bizarren Folgen: Unsere Berliner „Arbeitsgruppe für Elektrokardiologie“ sollte plötzlich in „Arbeitsgruppe plötzlicher Herztod“ umbenannt werden. Wir haben das verhindert. Es kann doch nicht sein, dass ein Patient an der Klinikpforte fragt: „Wie komme ich denn zum plötzlichen Herztod?“

Wenn weder die Registrierung spontaner Arrhythmien noch die Initiierung durch programmierte Stimulation einzeln und auch nicht kombiniert eine ausreichende Sicherheit für eine prognostische Aussage für den einzelnen Patienten liefern konnten, dann lag es nahe, nach weiteren Methoden einer Risikodetektion zu suchen. Belastungsuntersuchungen mittels Fahrradergometrie verbesserten die prognostische Vorhersage von ventrikulären Tachyarrhythmien nach Myokardinfarkt nicht [14]. Spätpotenziale [15], Herzfrequenzvariabilität [16], QT-Variabilität im Verlauf und -Dispersion, Barorezeptorsensitivität [17], T‑Wellen-Alternans [18], Hochfrequenzanalysen [19] und viele weitere Methoden wurden untersucht. Alle zeigten einzeln und in einigen Kombinationen [17, 20] manchmal sogar etwas deutlicher, dass eine Risikodetektion von Kammertachykardien in großen Patientenkohorten möglich ist. Auch genetische Untersuchungen und Blutspiegelbestimmung von Antiarrhythmika wurden hinzugenommen. Die Bestimmung biochemischer Marker [21] für chronische Entzündung (CrP, IL6, IL12, IL18, TNFα, LDL, Lp-PLA2), myokardialen (cTn, Osteopontin, ST2-Rezeptor, GDF15) oder oxydativen Stress (MPO, suPAR, PTX3, MMP, HSP70, Cystein, Glutathion), endokrine Untersuchungen (Aldosteron, Renin), weitere metabolische molekulare Biomarker (NEFA, ADMA) und neurohormonale Biomarker der Herzinsuffizienz (BNP, nt-proBNP, ADM, MR-proADM, Copeptin) haben diese Situation bis heute nicht grundsätzlich verändern können. Das Arrhythmierisiko des einzelnen Patienten blieb schwer vorhersagbar. Keine der Methoden allein, in keiner Kombination oder Kaskade hat es je geschafft, die Vorhersage eines arrhythmischen Ereignisses so abzusichern, dass die Implantation eines ICD gerechtfertigt erschien. Bis heute ist nur die linksventrikuläre Ejektionsfraktion in der Unterscheidung zwischen weniger und mehr gefährdeten Patienten akzeptiert.

Nach einem Vortrag zur Barorezeptorsensitivität in der Mitte der 90er Jahre hat Ludger Seipel, einer der Gründerväter der klinischen Elektrophysiologie auf der westlichen Elbseite, geäußert: „Sie machen das noch? Ich habe es schon längst aufgegeben.“

Letztlich zeigt der retrospektive Blick auf die Geschichte der klinischen Elektrophysiologie in der Beurteilung der ventrikulären Vulnerabilität, dass es möglich ist, die Wahrscheinlichkeit eines arrhythmischen Herztodes in einer definierten Patientengruppe vorherzusagen. Eine ausreichend sichere Prognose am einzelnen Patienten, die eine aggressive Therapie, z. B. Implantation eines ICD rechtfertigt, ist mit elektrophysiologischen Techniken jedoch nicht gelungen. Das selten ausgesprochene Argument war: „Wenn es uns nicht gelingt, auch nur die überwiegende Zahl der Gefährdeten zu erkennen, dann muss großzügiger implantiert werden, auch wenn dadurch zu viele wenig bedrohte Patienten einen ICD erhalten.“ Geblieben ist die prophylaktische Implantation bei hochgradig eingeschränkter linksventrikulärer Pumpfunktion, was einerseits alle Patienten mit Herzerkrankungen ausschließt, die eine weitgehend erhaltene Pumpfunktion aufweisen, als auch zu Implantationen bei vielen Patienten führt, die über viele Jahrzehnte keine adäquate Intervention erleben. Das paroxysmale Kammerflimmern bleibt bislang trotz vielfältiger diagnostischer Bemühungen der prognostischen Vorhersage ein im Einzelfall unerwarteter elektrischer Unfall, der nicht mit ausreichender Sicherheit vorherzusagen ist (Osman). Eigentlich ist es nach 40 Jahren elektrophysiologischer Diagnostik, heute oftmals ergänzt durch weitere diagnostische Parameter, eine paradoxe Erkenntnis: Der befürchtete elektrische Arrhythmieunfall ist mit allen elektrophysiologischen Techniken nicht über eine statistische Gruppenwahrscheinlichkeit hinaus abzusichern.
